# One-Tube Nested Real-Time PCR Assay for Rapid Screening of Porcine Cytomegalovirus in Clinical Samples

**DOI:** 10.3389/fvets.2020.586045

**Published:** 2020-10-28

**Authors:** Hye-young Wang, Joong Ki Song, Seongho Shin, Ki Myung Choi, Hyunil Kim

**Affiliations:** ^1^Optipharm, Inc., Cheongju, South Korea; ^2^Optipharm Animal Disease Diagnostic Center, Cheongju, South Korea

**Keywords:** porcine cytomegalovirus, one-tube nested real-time PCR, xenotransplantation, designated pathogen free pig, diagnosis

## Abstract

Porcine cytomegalovirus (PCMV) is a pathogen that must be removed from pigs for use as organ donors in xenotransplantation. Recently, it has been found that when donor pigs are infected with PCMV, a pig-to-non-human-primate xenotransplantation lower transplant survival by 2–3 times. Therefore, highly sensitive methods are needed to maintain designated pathogen free (DPF) pig and screen for xenografts. The purpose of this study was to evaluate the performance of commercially available method with one-tube nested real-time PCR assay to quickly detect PCMV infection in clinical samples and compare the results with those of sequence analysis. Molecular diagnostic methods were used to evaluate 127 samples, including tissues and blood samples from pigs suspected of PCMV infection. The detection rate for positive PCMV was 38.6% (*n* = 49), 23.6% (*n* = 30), and 12.6% (*n* = 16) in one-tube nested real-time PCR, nested PCR, and conventional PCR methods, respectively. All PCMV-positive samples in conventional PCR or nested PCR methods were also positive in the one-tube nested real-time PCR assay. All the PCR products in the three methods were checked for amplification of PCMV gene by PCR and subsequent direct sequencing. The results of one-tube nested real-time PCR were found to be consistent with those of sequence analysis for all the samples and showed good agreement (κ = 1). Our study found that the one-tube nested real-time PCR assay is more sensitive than the other two methods. This assay required approximately 1.5 h for completion. Therefore, we concluded that one-tube nested real-time PCR assay is a fast and reliable method for the characterizing pathogen responsible for PCMV infection.

## Introduction

In order to alleviate the shortage of human donor organs available for allograft, xenotransplantation using pig cells, tissues, or organs have been proposed (1–3). This may be related to the transmission of porcine mediated disease, so the maintenance of designated pathogen free pigs is required for xenotransplantation (4–6).

Porcine cytomegalovirus (PCMV), also known as Suid herpesvirus 2 (SuHV2), is an enveloped virus with a double-stranded linear DNA genome. PCMV can cause fever, reduced general condition, loss of appetite, numbness, neurological signs, and respiratory symptoms (e.g., sneezing, coughing, and dyspnea), acute to subacute disease, and high mortality and morbidity in piglets (3, 7). In a previous studies, the virus have been reported to be immunosuppressive pathogen primarily affecting the immune function of the macrophages and T lymphocytes, which can cause preclinical infection in adult pigs and reproductive failure in pregnant sows (7, 8). PCMV infection is widespread worldwide and has a high prevalence in swine herds. For this reason, many researchers have continued to pay attention to the potential risk to public health in interspecies transmission of PCMV and human xenotransplantation (7). In addition, international xenotransplantation association has a guideline that resource pig for xenotransplantation should be free from PCMV (9). These data indicate that it is essential to quickly detect PCMV infection with high sensitivity and specificity in the early stages of the resource swine herds.

So far, some molecular diagnostic methods, including polymerase chain reaction (PCR) (10–12), enzyme-linked immunosorbent assay (ELISA) (13, 14), loop-mediated isothermal amplification assay (LAMP) (15), and western blot analysis (16) for the detection of PCMV infection has been reported. These assays have low sensitivity, need to agarose gel analysis for amplification products, or have a risk of contamination, which can lead to incorrect results. In addition, companies such as Novateinbio and MyBioSource have ELISA products that detect antibody or antigen of PCMV, there are no commercialized products using molecular diagnostic methods until now. Real-time fluorescent quantitative PCR technology has become a powerful alternative platform for detection and differentiation of pathogenic viruses (3, 17, 18).

In this study, we developed a highly sensitive one-tube nested real-time PCR assay (Opti PCMV-qPCR; Optipharm, Osong, Republic of Korea) that combines nested PCR and real-time PCR to detect PCMV targeting DNA polymerase gene and consists of two sequential reactions in a single tube. The performance of the one-tube nested real-time PCR assay was evaluated using clinical samples suspected of PCMV compared to conventional PCR and nested PCR, and the results were confirmed by sequence analysis.

## Methods

### Preparation of DNA Samples

To evaluate the diagnostic performance of the PCR, nested PCR, and one-tube nested real-time PCR methods, a total of 127 field samples, including 37 lung tissues, 30 blood samples, 30 serums, and 30 feces samples, were provided by the Optipharm Animal Disease Diagnostic Center, which was commissioned from January to December 2019. In addition, 10 organs (lung, liver, pancreas, spleen, kidney, brain, heart, small intestine, nasal concha, and tonsil) of six pigs were analyzed to determine the infection rate of PCMV for each organ by one-tube nested real-time PCR assay. According to the manufacturer's recommendation, DNA was extracted from 200 μL of serum or 20 mg of organ tissue homogenate using a commercial automated system (Miracle-AutoXT Automated Nucleic Acid Extraction System, Intronbio, Seongnam, Republic of Korea). To avoid cross-contamination, all samples were individually processed and stored at −20°C. The content and purity of the extracted DNA was analyzed by measuring the absorbance at 260 and 280 nm by a spectrophotometer (Infinite 200 NanoQuant; Tecan, Switzerland).

### Conventional PCR and Nested PCR

To evaluate the usefulness of one-tube nested real-time PCR assay, the results of conventional PCR and nested PCR were compared. PCR primers for the conventional PCR and nested PCR used in this study were selected from the nucleotide sequence of the PCMV DNA polymerase gene determined by Hamel (10) and Fryer (11), respectively. PCR was performed using 20 μL of reaction mixture (Genetbio, Daejeon, Korea) containing 2 × master mix, 1 × primer mixture, 3 μL of sample DNA, and ddH_2_O added to achieve a final volume of 20 μL. The reaction conditions for conventional PCR and nested PCR using the outer primers (PCMVF1 and PCMVR1) were as follows: pre-denaturation at 94°C for 5 min; 40 cycles of 94°C for 30 s, 60°C for 30 s, 72°C for 30 s; and a final extension at 72°C for 10 min. For nested PCR using the inner primers (PCMVFB and PCMVR2), two microliters of the first-round PCR mixture was transferred to 20 μl of a premixed solution containing the PCR reagents at the same concentrations listed above. The amplification procedure was repeated for 40 cycles with the same time and temperature parameters as described above, except that annealing at 55°C for 30 s was used. The amplified target was visualized as a single band corresponding to a length of 413-bp for conventional PCR and 160-bp for nested PCR using the Chemi Doc system (Vilber Lourmat, Deutschland, Germany).

### One-Tube Nested Real-Time PCR Assay

**Figure 1:**
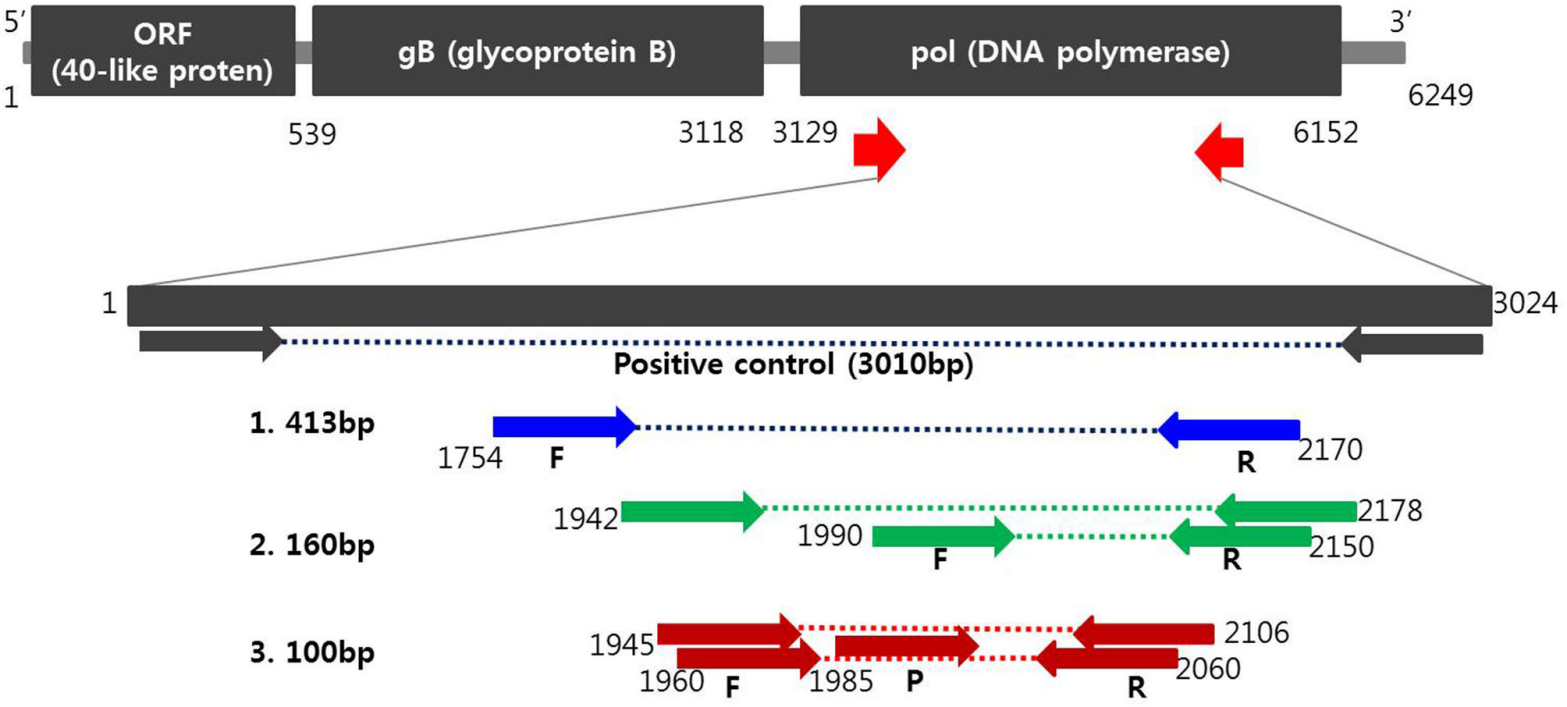
Schematic diagram of the DNA polymerase gene to distinguish PCMV from positions of primers and probes. A positive control was synthesized to verify the efficiency of the developed primers and probes. Primer sequences for conventional PCR and nested PCR were selected from the PCMV DNA polymerase gene determined by Hamel (1) and Fryer (2), respectively. Primers and probe sequences for one-tube nested real-time PCR (3) used in this study were selected at differed locations from the human and mouse cytomegalovirus DNA polymerase gene.

Oligonucleotide primers and probes corresponding to the two strands of the DNA polymerase gene of PCMV (Figure 1) were designed using Primer3Plus (http://www.bioinformatics.nl/cgi-bin/primer3plus/primer3plus.cgi). Primers were prepared as probes corresponding to the complementary strands and used exclusively thereafter. To verify the efficiency of the selected primers and probe, synthesized a positive control DNA sample (Bioneer, Daejeon, Republic of Korea) and amplified with custom PCR primers (forward, 5′-ATGACATTCTTAATCCATATAT-3′ and reverse, 5′-CACTGTCCCTAAAACTACTG-3′) resulting in amplicons of 3,010 bp. The resulting product was mutagenized after subcloning with pBHA vector. Detection of PCMV in clinical samples was performed with Opti PCMV real-time PCR (Optipharm), a quantitative one-tube nested real-time PCR-based assay, using a CFX-96 real-time PCR system (Bio-Rad, Hercules, CA, USA) for thermal cycling and fluorescence detection. Real-time PCR amplification was performed in a total reaction volume of 20 μL containing 10 μL of 2 × Thunderbird probe qPCR mix (Toyobo, Osaka, Japan), 2.5 μL of a mixture of 5 pmol each primers and 5 pmol TaqMan probe that were labeled with fluorophores (FAM-BHQ1), and 3 μL template DNA. The real-time PCR kit consisted of an internal control (IC) DNA and a primer set for IC DNA amplification included in the reaction mixture, which was used to indicate successful nucleic acid extraction, sample quality, and to confirm the presence of PCR inhibitors in the reaction. Therefore, it does not compete directly with the amplification of species–specific targets in multiplex real-time PCR. Positive (plasmid PCMV DNA) and negative controls consisting of molecular grade (DNAse/RNAse-free) water (Ultra pure water; Welgene, Gyeongsan, Republic of Korea) without template DNA were included in each assay. The assay was carried out under the following conditions: 95°C for 3 min, then 10 cycles of 3 s at 95°C and 30 s at 60°C, and then by 40 cycles of 3 s at 95°C and 30 s at 55°C. Each sample was tested in duplicate by running the PCR cycle twice and a positive result was obtained when the C_T_ value was <35.

### Interfering Reactions and Reproducibility Analysis

For interfering reactions, we used the following 7 substances by concentration: EDTA and sodium citrate (1, 10, 20, and 50 mM), and heparin (250, 300, 375, and 500 IU) for anticoagulants, phosphate buffered saline (PBS; 1, 5, 10, and 20X) for tissue emulsion, EtOH and xylene (1, 5, 10, 20, and 50%), and blood (1, 5, and 10%). The repeatability and reproducibility of this assay were performed with a total of 240 tests (10 days × 2 runs/day × 4 replicates × 3 lots). The coefficient of variation (CV) was calculated according to the form of the mean C_T_ values/standard deviation (SD).

### Sequence Analysis

To confirm the results of the three molecular diagnostic methods, PCR amplicons of all clinical isolates were sequenced using an ABI 3730 automated DNA sequencer (Applied Biosystems, Foster City, CA, USA) and the ABI Prism BigDye Terminator (Applied Biosystems) system (CosmoGenetech, Republic of Korea). The primer sets used to amplify the target DNA polymerase gene were 5′-CCTGATCTTAAATGACGAGGACGTGAC-3′ (413F) and 5′-ACCGTCTGAGAGACTGAACTTCTCTGACAC-3′ (413R), 5′-AGGACCCTATGTTGGCAYTGATAC-3′ (1945F) and 5′-TCGTCTGCCTRAGCATGTCC-3′ (2106R), which resulted in a 413-bp and 162-bp PCR product, respectively. The obtained sequence was compared with that of the National Center for Biotechnology Information GenBank database. The primer sequences had been removed from alignment sequences before phylogenetic analysis. Multiple alignments of nucleotide sequences based on the PCR product of the PCMV DNA polymerase gene mentioned above were performed using MUSCLE within the Phylogeny.fr software (19). Then, Gblocks was used as a collection method to align the sequences, assess the phylogenetic relationships by the PhyML using the 49 strains isolated in this study, together with 12 PCMV strains deposited in the GenBank database, and finally visualized the phylogenetic tree by TreeDyn.

## Results

### Analytical Sensitivity and Specificity of One-Tube Nested Real-Time PCR

**Figure 2:**
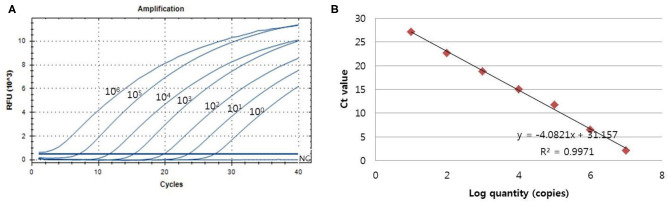
The detection limit of the one-tube nested real-time PCR assay was evaluated using 10-fold serial diluted samples. Serially diluted PCMV control DNA ranging from 10^6^ copies to 1 copy per reaction were used to determine the detection limit of the one-tube nested real-time PCR assay. In the one-tube nested real-time PCR assay, the amplification curve of the specific probe **(A)** for detecting PCMV is shown. The overall detection limit of this assay for the PCMV ranged from approximately 1 copy DNA per reaction. C_T_ was plotted against the input of the quantity of PCMV DNA (repeated 20 times). The linearity **(B)** was generated by plotting the log quantity of PCMV DNA vs. the corresponding C_T_ value, and the coefficients of determination of the linear regression were 0.997 with a slope of −4.082. The fluorescence intensity is displayed on the Y-axis (*R*^2^ = reporter signal/passive reference signal). RFU, relative fluorescence unit; *R*^2^, fluorescence units.

The analytical sensitivity of the assay for PCMV detection was determined using a standard curve that 10-fold serially diluted (10^6^ copies−1 copy) of plasmid DNA containing cloned PCMV gene (Figure 2). The sensitivity was estimated as the lowest PCMV gene copies yielding a positive result in all 20 replicates, and the corresponding C_T_ value were selected as the analysis cutoff. A standard curve was generated by plotting the log quantity of PCMV DNA vs. the corresponding C_T_ value, and the coefficient of determination (*R*^2^) for linear regression was 0.997 with a slope of −4.082. The detection limit of the one-tube nested real-time PCR assay for PCMV was detected at a concentration of 1 copy per reaction. The C_T_ values of PCMV DNA concentration ranged from 1.2 to 28.5, and mean C_T_ values were 2.1 ± 2.2 (95% confidence interval [CI], 1.2–3) to 27.1 ± 1.7 (95% CI, 26.4–27.8) and the CV was <3%.

To assess the potential cross-reactivity, analytical specificity was performed with 40 samples with concentrations above 10^4^ copies of individual bacterial/viral genes. The one-tube nested real-time PCR assay to detect PCMV-positive showed negative results in all strains except control PCMV. Hence, these primers and probes did not react with any bacterial and viral strains (Table 1).

**Table 1:** Analytical specificity of the one-tube nested real-time PCR assay to detect PCMV with 40 strains.

**No**.	**Species**	**Isolate**	**Sample type**	**One-tube nested real-time PCR**	
				**PCMV (Ct)**	**IC (Ct)**
1	*Porcine reproductive and respiratory syndrome virus*	Field isolate	Tissue	N/A	20.58
2	*Porcine reproductive and respiratory syndrome virus*	Field isolate	Tissue	N/A	21.46
3	*Porcine reproductive and respiratory syndrome virus*	Field isolate	Tissue	N/A	20.38
4	*Porcine reproductive and respiratory syndrome virus*	Field isolate	Tissue	N/A	20.54
5	*Swine Influenza virus*	Field isolate	Tissue	N/A	19.72
6	*Hemophilus parasuis*	Field isolate	Tissue	N/A	18.94
7	*Hemophilus parasuis*	Field isolate	Tissue	N/A	20.7
8	*Mycoplasma hyopneumoniae*	Field isolate	Tissue	N/A	22.2
9	*Mycoplasma hyopneumoniae*	Field isolate	Tissue	N/A	20
10	*Mycoplasma hyopneumoniae*	Field isolate	Tissue	N/A	22.28
11	*Porcine rotavirus*	Field isolate	Tissue	N/A	20.66
12	*Porcine rotavirus*	Field isolate	Stool	N/A	20.23
13	*Actinobacillus pleuropneumoniae*	Field isolate	Tissue	N/A	20.31
14	*Porcine epidemic diarrhea virus*	Field isolate	Stool	N/A	21.56
15	*Porcine epidemic diarrhea virus*	Field isolate	Sludge	N/A	20.46
16	*Porcine Parvovirus*	Field isolate	Collagen	N/A	22.21
17	*Porcine Parvovirus*	Field isolate	Collagen	N/A	21.13
18	*Porcine circovirus type 2*	Field isolate	Tissue	N/A	21.07
19	*Porcine circovirus type 2*	Field isolate	Tissue	N/A	21.1
20	*Porcine circovirus type 2*	Field isolate	Tissue	N/A	22.78
21	*Porcine circovirus type 2*	Field isolate	Tissue	N/A	21.19
22	*Porcine circovirus type 3*	Field isolate	Tissue	N/A	22.32
23	*Porcine circovirus type 3*	Field isolate	Tissue	N/A	21.41
24	*Porcine circovirus type 3*	Field isolate	Tissue	N/A	20.11
25	*Porcine circovirus type 3*	Field isolate	Tissue	N/A	20.84
26	*Porcine circovirus type 3*	Field isolate	Tissue	N/A	19.28
27	*Porcine circovirus type 3*	Field isolate	Tissue	N/A	22.45
28	*Escherichia coli*	ATCC 25922	Culture	N/A	21.33
29	*Escherichia coli*	ATCC 35150	Culture	N/A	21.1
30	*Salmonella enteritidis*	ATCC 13076	Culture	N/A	19.36
31	*Salmonella typhi*	ATCC 19430	Culture	N/A	21.13
32	*Salmonella paratyphi*	ATCC BAA-1250	Culture	N/A	20.66
33	*Salmonella Newport*	ATCC 6962	Culture	N/A	20.08
34	*Salmonella typhimurium*	ATCC 14028	Culture	N/A	18.58
35	*Staphylococcus aureus*	ATCC 25923	Culture	N/A	21.18
36	*Staphylococcus aureus*	ATCC 29213	Culture	N/A	21.55
37	*Staphylococcus aureus*	ATCC 6538	Culture	N/A	21.49
38	*Clostridium perfringens*	ATCC 13124	Culture	5.13	21.66
39	*Toxoplasma gondii*	ATCC 50853	Culture	10.74	21.9
40	*Bordetella bronchiseptica*	ATCC 4617	Culture	N/A	20.61
41	*Porcine cytomegalovirus*	PC	Culture0	10.38	20.24
42	*Porcine cytomegalovirus*	PC	Culture	10.74	20.61
43	NC	-	-	N/A	19.8

*ATCC, American Type Culture Collection; PCMV, porcine cytomegalovirus*.

### Results of Interfering Reactions and Reproducibility Analysis by One-Tube Nested Real-Time PCR

We performed the interference reaction with 7 substances by concentration. As a result, there was no interference below 50 mM EDTA, 50 mM sodium, 375IU heparin, 10X PBS, 50% EtOH, 50% xylene, and 10% blood (data not shown). For repeatability and reproducibility, the measured number for the 3 concentrations of positive control was 240 (10 days × 2 runs/day × 4 replicates × 3 lots). As shown in Table 2, the CV for intra- and inter-assay variability ranged from 0.6 to 2.5% and 1.2 to 2.5%, respectively, which were all <3%. The intra-laboratory reproducibility results at cutoff concentration (about 1 copy) over time were 96.4% (κ = 0.96, 95% CI, 0.926–0.985). Based on the experimental results, we suggest that this assay may have stable results.

**Table 2:** Results of intra- and inter-assay for repeatability and reproducibility analysis.

**Copies/*μℓ***	***N***	**Total**			**Intra-assay**			**Inter-assay**					
					**Within-run**			**Between-run**			**Between-day**		
		**C_**T**_ avg**	**SD**	**CV(%)**	**C_**T**_ avg**	**SD**	**CV(%)**	**C_**T**_ avg**	**SD**	**CV(%)**	**C_**T**_ avg**	**SD**	**CV(%)**
10^5^	240	7.7	0.0	0.5	7.7	0.1	0.8	7.7	0.1	1.9	7.7	0.1	1.3
10^3^	240	15.6	0.1	0.6	15.6	0.1	0.6	15.7	0.3	1.8	15.6	0.2	1.2
10^1^	240	23.8	0.1	0.3	23.9	0.6	2.5	23.8	0.5	2.0	23.6	0.6	2.5

*N, test number of inter- and intra- assay; avg, average; SD, standard deviation; CV, coefficients of variation*.

### Detection of PCMV Using Conventional PCR, Nested PCR, and One-Tube Nested Real-Time PCR Methods in Clinical Samples

**Figure 3:**
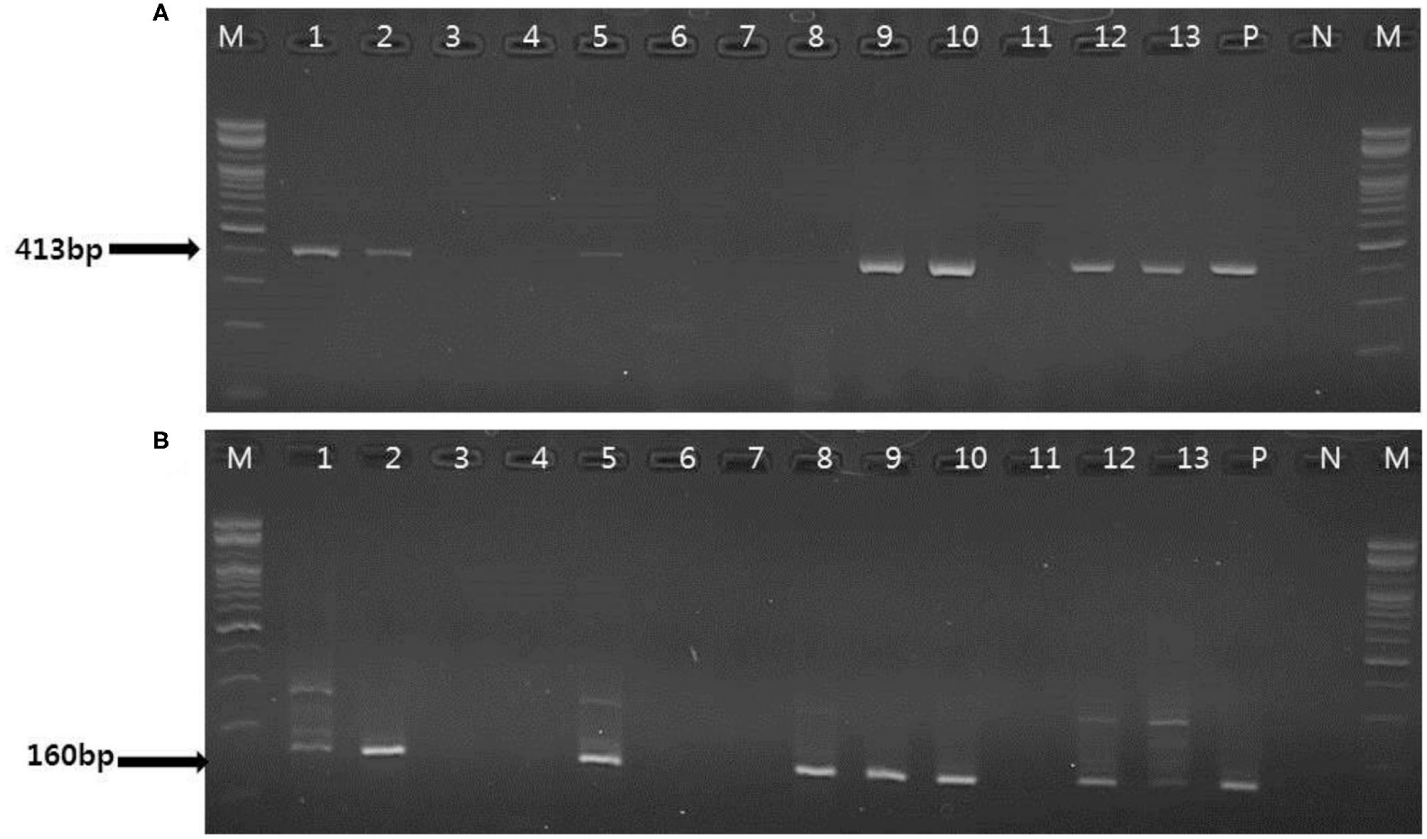
Representative results of the conventional PCR and nested PCR with clinical samples. **(A)** Conventional PCR results. Lanes 1, 2, 5, 9, 10, 12–13: positive; M, 100 bp DNA ladder (Bioneer); P, positive control; N, negative control. **(B)** Nested PCR results. Lane 1, 2 5, 8–10, 12–13: positive; M, 100 bp DNA ladder (Bioneer); P, positive control; N, negative control.

To evaluate the performance of one-tube nested real-time PCR assay, a total of 127 clinical samples, including lung tissues (*n* = 37, 29.1%), whole bloods (*n* = 30, 23.6%), serums (*n* = 30, 23.6%), and feces (*n* = 30, 23.6%), were used. The results were compared with those of conventional PCR (Figure 3A) and nested PCR (Figure 3B). Of the 127 clinical samples, 49 (38.6%) samples were positive for PCMV, while 78 (61.4%) samples were negative as detected by one-tube nested real-time PCR. On the other hand, 16 (12.6%) and 30 (23.6%) were detected by conventional PCR and nested PCR, respectively (Table 3). All clinical samples showed positive IC signals, and the C_T_ values of the 49 positive and 81 negative samples ranged from 17.6 to 22.5 (mean 20.6, SD ± 1) and 17.7 to 22.5 (mean 20.9, SD ± 0.8), respectively. In addition, the C_T_ values of PCMV-positive samples ranged from 14 to 28.8 (mean 22.8, SD ± 4.1). In a pilot study, we investigated detection for PCMV infection in 10 organs (lung, liver, pancreas, spleen, kidney, brain, heart, small intestine, nasal concha, and tonsil) of 6 pigs. As a result, the organs with the most prevalent PCMV detected were lung, spleen, and nasal concha (100%), followed by liver, small intestine, and tonsil (83.3%), kidney, heart, and pancreas (66.7%), and brain (50%), respectively (data not shown).

**Table 3:** Detection of PCMV DNA in 127 clinical samples suspected of PCMV infection using the conventional PCR, nested PCR, and one-tube nested real-time PCR assay.

**Sample**	**Total no. (%) of samples**	**Detection of PCMV, no. (%) of isolates**							
		**Conventional PCR**		**Nested PCR**		**One-tube nested real-time PCR**			
		**Positive (%)**	**Negative (%)**	**Positive (%)**	**Negative (%)**	**Positive (%)**	**Negative (%)**	**IC ranged C_**T**_ value (mean ± SD)**	**PCMV ranged C_**T**_ value (mean ± SD)**
Tissue	37 (29.1)	16 (43.2)	21 (56.8)	25 (67.6)	12 (32.4)	32 (86.5)	5 (13.5)	17.6–22.1 (20.5 ± 0.9)	14–27.3 (21.1 ± 3.9)
Blood	30 (23.6)	0 (0)	30 (100)	1 (3.3)	29 (96.7)	6 (20)	24 (80)	19.3–21.7 (21.1 ± 0.5)	23.1–28.1 (26.6 ± 1.8)
Serum	30 (23.6)	0 (0)	30 (100)	2 (6.7)	28 (93.3)	6 (20)	24 (80)	20.5–22.5 (21.6 ± 0.5)	20–28.5 (21.6 ± 0.5)
Feces	30 (23.6)	0 (0)	30 (100)	2 (6.7)	28 (93.3)	5 (16.7)	25 (83.3)	17.7–21.2 (20.1 ± 0.9)	25.4–28.8 (27.3 ± 1.4)
Total	127 (100)	16 (12.6)	111 (87.4)	30 (23.6)	97 (76.4)	49 (38.6)	78 (61.4)	17.6–22.5 (20.8 ± 0.9)	14–28.8 (22.8 ± 4.1)

*PCMV, porcine cytomegalovirus; IC, internal control; SD, standard deviation*.

### Comparison of the Results Between the One-Tube Nested Real-Time PCR Assay and Sequence Analysis for the Detection of PCMV in Clinical Samples

**Figure 4:**
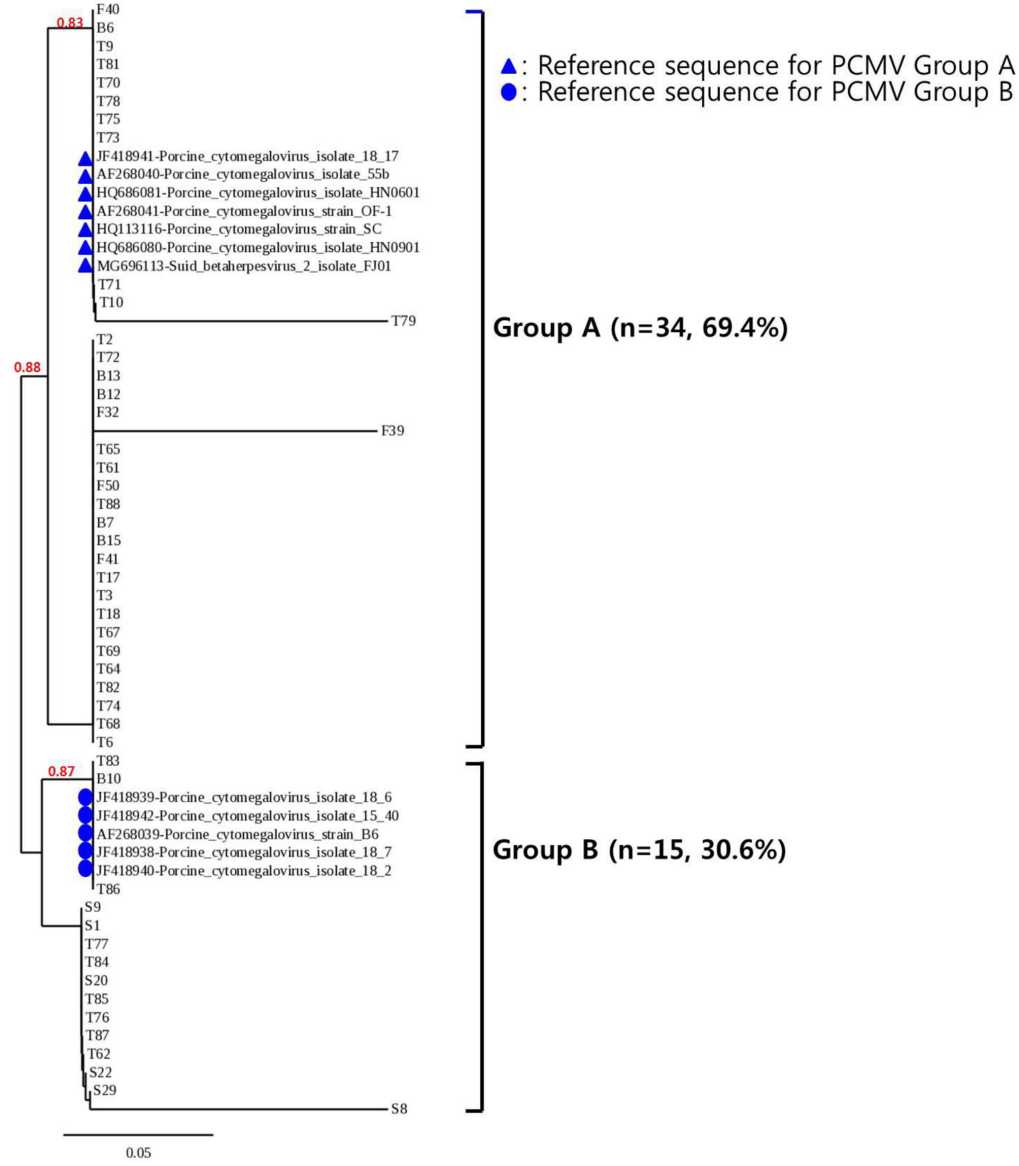
Phylogenetic analysis of 49 PCMV isolates. The phylogenetic tree was constructed using Phylogeny.fr software after the alignment of the 49 sequenced results. The Phylogeny.fr software offers MUSCLE for multiple sequence alignments, Gblocks for alignment curation, PhyML for phylogenetic reconstruction, and TreeDyn for graphical representation of trees. Analysis of phylogenetic tree showed that the sequences obtained from the one-tube nested real-time PCR assay were similar to those of the reference strains FJ01 and B6, with two divided groups (Groups A and B) accounting for 69.4% (*n* = 34) and 30.6% (*n* = 15), respectively.

To confirm the results obtained from the one-tube nested real-time PCR assay, sequence analysis was performed using the same clinical samples. All 49 samples detected as PCMV-positive by one-tube nested PCR assay were consistent with the sequencing results. Our study showed that nested PCR had a higher positive rate than conventional PCR (23.6% vs. 12.6%), but the one-tube nested real-time PCR assay (38.6%) was more sensitive than the other two methods. The agreement rate between one-tube nested real-time PCR assay and conventional PCR or nested PCR was 74% (95% CI 0.654–0.813, *p* < 0.001) and 85% (95% CI 0.776–0.907, *p* < 0.001), respectively (Table 4). In addition, the agreement rate of the one-tube nested real-time PCR assay and sequence analysis was 100% (95% CI 0.976–1.000, *p* < 0.001). Using sequence analysis, the sensitivity, specificity, and positive and negative predictive values of PCMV results by one-tube nested real-time PCR assay were 100% (*n* = 49, 95% CI 0.947–1.000, *p* < 0.001), 100% (*n* = 78, 95% CI 0.959–1.000, *p* < 0.001), 100% (95% CI 0.947–1.000, *p* < 0.001), 100% (95% CI 0.959–1.000, *p* < 0.001), respectively. All PCMV-positive samples in conventional PCR or nested PCR methods were also positive in the one-tube nested real-time PCR assay. The phylogenetic tree was constructed using Phylogeny.fr software (19) after alignment of the 49 sequenced results. The 49 sequences obtained from direct sequence analysis of clinical samples were found that 69.4% (*n* = 34) in the FJ01 strain (Groups A; accession no. MG696113) groups and 30.6% (*n* = 15) in the B6 strain groups (Groups B; accession no. AF268039) were similar (Figure 4).

**Table 4:** Clinical sensitivity and specificity between the one-tube nested real-time PCR assay and conventional PCR, nested PCR, and sequence analysis methods stratified by PCMV suspected samples.

**Molecular assays**	**One-tube nested real-time PCR**		**Sensitivity, % (*n*) (95% CI)**	**Specificity, % (*n*) (95% CI)**	**PPV, % (*n*) (95% CI)**	**NPV, % (*n*) (95% CI)**	**Agreement, % (*n*) (95% CI)**	**κ coefficient (95% CI)**
	**Positive**	**Negative**						
**Conventional PCR**								
Positive	16	0	32.7 (16/49) (0.199–0.475)	100 (78/78) (0.959–1.000)	100 (16/16) (0.829–1.000)	70.3 (78/111) (0.608–0.785)	74 (94/127) (0.654–0.813)	0.373 (0.1893–0.5573)
Negative	33	78						
**Nested PCR**								
Positive	30	0	61.2 (30/49) (0.462–0.748)	100 (78/78) (0.959–1.000)	100 (30/30) (0.905–1.000)	80.4 (78/97) (0.711–0.877)	85 (108/127) (0.776–0.907)	0.659 (0.518–0.800)
Negative	19	78						
**Sequence analysis**								
Positive	49	0	100 (49/49) (0.947–1.000)	100 (78/78) (0.959–1.000)	100 (49/49) (0.947–1.000)	100 (78/78) (0.959–1.000)	100 (127/127) (0.976–1.000)	1 (0.963–1.000)
Negative	0	78						

*PPV, positive predictive value; NPV, negative predictive value; 95% CI, 95% confidence interval*.

## Discussion

Pigs are frequently infected with PCMV, but infected adult animals do not always show symptoms of disease. Even though the virus remains latent, it can be transmitted the virus to anyone who receives a swine transplantation. Recently, pig-to-non-human-primate xenotransplantation have shown that transplant survival rates are 2–3 time lower when donor pigs were infected with PCMV (4). Therefore, highly sensitive methods are needed to select PCMV-free pigs and to screen for xenografts. The purpose of this study was to evaluate the analytical performance and clinical efficacy of newly developed high-sensitivity one-tube nested real-time PCR assay, taking advantage of conventional PCR and nested PCR for fast and accurate detection based on the DNA polymerase gene of PCMV. One-tube nested real-time PCR is a simple and sensitive method for the detection and identification of PCMV through sequential amplification of the DNA polymerase gene sequence of PCMV in a single tube (20). One-tube nested real-time PCR is about 100 times more sensitive than conventional PCR or nested PCR. Our results are consistent with previous reports indicated that nested PCR improved sensitivity significantly compared to conventional PCR, mostly due to two sequential amplification steps of the target gene (21–23). In addition, one-tube nested real-time PCR has the same advantages as conventional real-time PCR, including ease, rapidity (turnaround time of 1.5 h), accuracy, low risk of cross-contamination due to sequential reactions in a single tube, reproducibility, and high-throughput capabilities allows quick screening of multiple samples (24, 25). Moreover, the development of fluorescence-based real-time monitoring allows PCR to be a quantitative method, and PCR amplification or quantification was performed in the same reaction tube to reduce possible errors. Owing to its high sensitivity, one-tube nested real-time PCR has been proposed as an excellent method for determining various diseases (24, 25). In Korea, performance evaluation data such as sensitivity, specificity, reproducibility, and repeatability are required for product approval. To evaluate the performance and accuracy of the one-tube nested real-time PCR assay, the synthesized PCMV plasmid DNA was used as a reference standard, and C_T_ values were repeatedly measured 20 times. The detection limit of the assay was determined to be 1 copy per reaction. The assay was tested using various strains, no cross-reactivity between strains was observed, and the presence of other samples in PCMV-infected strains was demonstrated to not affect the performance of the assay.

The DNA polymerase gene used in this study was proven to be a highly conserved sequence with no significant variation among several PCMV isolates (3), and showed a difference of 72.7 and 68.9% compared to that of human or mouse DNA polymerase genes, respectively. Nevertheless, the positive rate was high in samples from tissue other than sites. Thus, studies on areas with high PCMV infection in each organ should be conducted to gain further insights into xenotransplantation.

## Conclusions

The one-tube nested real-time PCR assay generally showed high agreement and specificity with sequence analysis. This assay is a fast, accurate, and convenient tool for simultaneously detecting the presence of PCMV infection in many samples. Therefore, using the newly developed molecular diagnostic assay in PCMV screening can help detect the most important disease, while reducing false-positives or false-negatives. It is also likely to be used as a sensitive and specific tool for early detection and diagnosis of PCMV infection.

## Data Availability Statement

The datasets presented in this study can be found in the GenBank with accession code AF268039.

## Author Contributions

H-yW performed evaluation of the experiments, analyzed the data, and drafted the manuscript. JS and SS provided clinical samples and clinical information. KC and HK revised the manuscript. All authors have read and approved the final manuscript.

## Conflict of Interest

All authors were employed by Optipharm, Inc. The authors declare that the research was conducted in the absence of any other commercial or financial relationships that could be construed as a potential conflict of interest.
